# Fire-Exposed Fly-Ash-Based Geopolymer Concrete: Effects of Burning Temperature on Mechanical and Microstructural Properties

**DOI:** 10.3390/ma15051884

**Published:** 2022-03-03

**Authors:** Siti Nooriza Abd Razak, Nasir Shafiq, Laurent Guillaumat, Syed Ahmad Farhan, Vicky Kumar Lohana

**Affiliations:** 1 Department of Civil and Environmental Engineering, Universiti Teknologi PETRONAS, Seri Iskandar 32610, Malaysia; nasirshafiq@utp.edu.my (N.S.); vicky_19000193@utp.edu.my (V.K.L.); 2 Laboratoire Angevin de Mécanique, Procédés et Innovation, École Nationale Supérieure d’Arts et Métiers, 49035 Angers, France; laurent.guillaumat@ensam.eu; 3 Institute of Self-Sustainable Building for Smart Living, Universiti Teknologi PETRONAS, Seri Iskandar 32610, Malaysia; syed.af_g02626@utp.edu.my; 4 Department of Civil Engineering, Indian Institute of Technology Madras, Chennai 600036, India

**Keywords:** compressive strength, fire resistance, fly ash, Fourier-transform infrared spectroscopy, geopolymer concrete, mass loss, scanning electron microscopy

## Abstract

Geopolymer concrete possesses superior fire resistance compared to ordinary Portland cement (OPC)-based concrete; however, there are concerns regarding its vulnerability when exposed to real fire events. In the present study, the fire resistance of fly-ash-based geopolymer concrete was evaluated relative to that of OPC-based concrete. Concrete specimens of standard strength grades of 20, 40, and 60 MPa were exposed to fire at 500 and 1200 °C for 2 h to simulate real fire events. Visual observation was performed, mass loss and residual compressive strength were measured, and scanning electron microscopy (SEM) and Fourier-transform infrared spectroscopy (FTIR) analyses were conducted. OPC-based concrete suffered major cracks accompanied with spalling for the high-strength specimen, while geopolymer concrete experienced minor cracks with no spalling. Mass losses of the geopolymer concrete—of 1.69% and 4%, after the exposure to fire at 500 and 1200 °C, respectively—were lower than those of the OPC-based concrete. More than 50% of the residual compressive strength for low- and medium-strength geopolymer concrete, after the exposure to fire at 1200 °C, was maintained. After the exposure to fire at 500 °C, the residual compressive strength of the geopolymer concrete increased from 13 to 45%, while the OPC-based concrete was not able to sustain its compressive strength. SEM images showed that the matrix of the geopolymer concrete, after the exposure to fire, was denser than that of the OPC-based concrete, while the FTIR spectra of the geopolymer concrete showed a minor shift in wavelength. Hence, our findings indicate that fly-ash-based geopolymer concrete has an excellent fire resistance as compared to OPC-based concrete.

## 1. Introduction

At present, fire events that are caused by the ignition or explosion of hydrocarbon-based fuels in the built environment are common. These events induce a rapid rise in temperature from the flashover state to more than 1100 °C within a few minutes [1,2]. In light of the occurrence of destructive fire events, fire resistance has become one of the most important properties of construction materials. Ultimately, there is not a single material that cannot be destroyed by the effects of heat. Nevertheless, fire-resistant materials can be treated to reinforce them against extreme temperatures; their susceptibility to fire can be massively reduced by fireproofing [3].

As the most utilized material in the construction industry, concrete is generally known to have good fire resistance. Most studies involving ordinary Portland cement (OPC)-based concrete emphasize thermal properties and the addition of reinforcement to improve fire resistance; however, the concrete still cannot withstand high temperatures. The properties of concrete at the physical and chemical levels are adversely affected by fire. In the case of destructive fires, even high-quality designed concrete structures are severely affected, and eventually collapse. In 2017, the 24-storey Grenfell Tower in London, England caught fire, which was started by a malfunctioning fridge–freezer on the fourth floor; the event caused 72 deaths and severe structural damage [4]. 

Geopolymer concrete has been advocated as an eco-friendly alternative to OPC-based concrete. Davidovits [5] propounded the premise that strength of geopolymer concrete can increase after exposure to high temperatures, owing to the geopolymerization process. Kong et al. [6] reported that the residual compressive strength of fly-ash-based geopolymer paste increased by 6% after exposure to 800 °C, and explained that, throughout the heating process, the high permeability of the material contributed towards reducing the damage by facilitating the escape of moisture from within the matrix. Sintering increased the strength of the concrete at 800 °C. An increase in strength by a similar amount was also mentioned by Pan et al. [7] in geopolymer mortar, where no spalling and cracks were found on the surface. On the other hand, Aslani [8] also stressed that the strength of geopolymer concrete that contain coarse aggregates decreased owing to the incompatibility between the aggregates and the binder.

Even though geopolymer concrete possesses superior fire resistance compared to OPC-based concrete, there are still concerns regarding its vulnerability when exposed to real fire events; its fire resistance has not been comprehensively tested in previous studies, which have predominantly adopted exposure to fire in controlled environments that differ to real fire events. Therefore, the present study evaluates the fire resistance of fly-ash-based geopolymer concrete that was exposed to fire at 500 and 1200 °C for 2 h to simulate real fire events; its fire resistance was then compared with that of OPC-based concrete.

## 2. Methodology

Trial mixes were produced to examine the fire resistance of fly-ash-based geopolymer concrete with different grades of strength when exposed to fire at 500 and 1200 °C. The mixes were produced to meet the requirements of standard strength grades of 20, 40, and 60 MPa. OPC-based concrete specimens were employed as control specimens in order to comparatively evaluate the fire resistance of the geopolymer concrete. 

In total, 12 cube specimens of geopolymer and OPC-based concrete of a standard size of 100 mm × 100 mm × 100 mm were prepared based on the optimal mix design. The specimens were exposed to fire at 500 and 1200 °C for 2 h. The transfer of heat within the specimens was recorded using K-type thermocouples. Visual observation of the physical appearance of the concrete, after cooling, was performed in order to detect cracking and spalling during exposure to the fire. Mass losses were determined by weighing the specimens before and after exposure to the fire. Compression tests were conducted to determine their residual compressive strength. Scanning electron microscopy (SEM), with gold coating and an accelerating voltage of 5 kV, at working distance ranges from 4 to 10 mm, was conducted in order to analyze the morphology of the specimens. The SEM was conducted using a field emission scanning electron microscope of model SUPRA™ 66VP, which was manufactured by ZEISS International (Jena, Germany). Fourier-transform infrared (FTIR) spectroscopy was performed to analyze the changes in microstructural properties as a result of the exposure to the fire. The FTIR was conducted using an FTIR spectrometer of model Frontier™ 01, which was manufactured by PerkinElmer (Akron, OH, USA).

### 2.1. Materials

Commercially available Class F fly ash that meets the requirements of ASTM C618-19 [9] was employed to produce the geopolymer concrete specimens. X-ray fluorescence (XRF) analysis was conducted using an XRF spectrometer of model S8 TIGER, which was manufactured by Bruker AXS (Karlsruhe, Germany) to determine the elemental compositions of the OPC and fly ash employed in the present study, which are shown in Table 1. Loss on ignition, as shown in Table 1, refers to the loss in weight as a result of heating a sample of the material to a high temperature during the analysis. 

**Figure 1:**
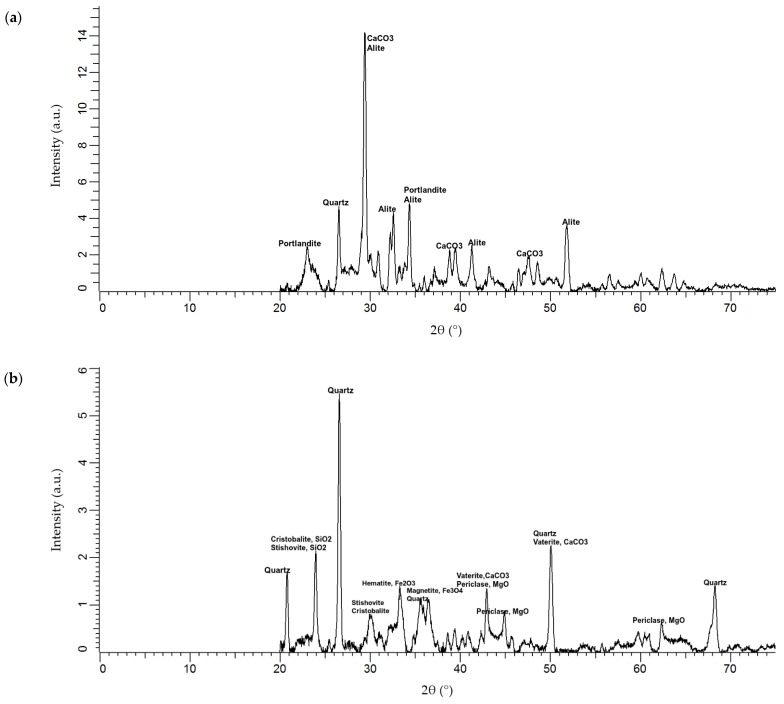
Mineralogy of the (**a**) OPC and (**b**) fly ash employed.

Figure 1 displays the mineralogy of the OPC and fly ash, which was ascertained via X-ray diffraction (XRD) analysis using an X-ray diffractometer of model X’Pert^3^ Powder, which was manufactured by Malvern Panalytical B.V. (Almelo, The Netherlands). As per the diffraction peaks for the phases in the 28.5° 2-θ to 30° 2-θ range, the OPC exhibited comparatively high crystallization phases consisting of alite and calcium carbonate content. Broad diffraction corresponding to portlandite was also detected in the 22° 2-θ to 25° 2-θ range, indicating that the fly ash was mostly glassy, with high crystallization for phases of quartz, cristobalite, and stishovite. The quartz phase showed high crystallinity in the 20° 2-θ, 26° to 27° 2-θ, and 50° 2-θ ranges. The cristobalite and stishovite showed a high peak of crystallization phase in the 24° 2-θ range. The identified phases belong to the silica group, which is the key component of pozzolanic characteristics, and can generate cementitious compounds in the presence of an alkaline solution.

Sodium hydroxide and sodium silicate were employed as the alkaline activators. The sodium silicate solution was of a standard grade, with a SiO_2_/Na_2_O weight ratio of 2.5, a density of 1.39 g/mL, and a temperature of 25 °C. For the sodium hydroxide, a 10 M concentration was adopted. The sodium hydroxide solution was prepared by dissolving sodium hydroxide pellets in distilled water for ~24 h prior to preparing the specimens; 40 g of the sodium hydroxide was dissolved in 1 L of water to produce 1 M concentration of sodium hydroxide. Hence, in order to produce 10 M concentration of sodium hydroxide, 400 g of sodium hydroxide pellets was dissolved in 1 L of water.

### 2.2. Casting and Curing

Table 2 shows the concrete mix proportions for casting of the specimens with the standard strength grades of 20, 40, and 60 MPa for the OPC-based concrete (OPC20, OPC40, and OPC60) and geopolymer concrete (GEO20, GEO40, and GEO60) [10,11]. 

For preparation of the OPC-based concrete mix, sand and coarse aggregates were mixed for 30 s, before adding half of the water, and then mixing again for a minute. Mixing was then stopped for 8 min to allow the sand and coarse aggregates to absorb some water prior to adding OPC. After eight minutes, OPC was added, and mixing was continued for one minute. The remaining half of the water was then added, and mixing was continued again for one more minute. 

For preparation of the geopolymer concrete mixes, sand and coarse aggregates were mixed for 30 s prior to adding fly ash, and then mixed for a further minute, ensuring that the constituents were uniformly mixed. Subsequently, sodium hydroxide and sodium silicate were stirred into the mixture for three minutes, ensuring that they were uniformly mixed without segregation.

The fresh mixtures were then poured into the mold in two layers, with each layer tamped using a rod to remove trapped air. Cast specimens were cured at ambient temperature for 24 h to allow them to harden. OPC-based concrete specimens were taken out of the mold and cured in water for 28 days [11]. On the other hand, geopolymer concrete specimens were exposed to heat curing for 24 h at 60 °C, and then left to cool at room temperature for 28 days prior to performing flame tests [10].

### 2.3. Flame Tests

**Figure 2:**
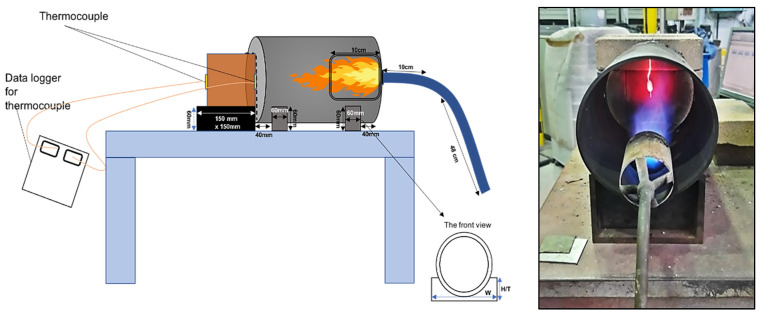
Flame test setup.

A setup using natural gas was established to conduct flame tests in order to evaluate the fire resistance of the specimens when subjected to fire at 500 and 1200 °C for 2 h. For OPC-based concrete, the specimens were taken out of the curing tank and dried for 1 h, or until the surface of the concrete has dried, prior to the test. The distance between the fire torch and the specimen was kept constant at 200 mm, ensuring that the flame did not damage the thermocouple. Prior to commencing the test, the specimens were weighed to ensure that their density was consistent. The apparatus for the flame test was set up as shown in Figure 2. Thermocouples were placed in front of the exposed surface of the concrete and connected to a data logger to record the temperature obtained from the fire.

## 3. Results and Discussion

### 3.1. Visual Observation

**Figure 3:**
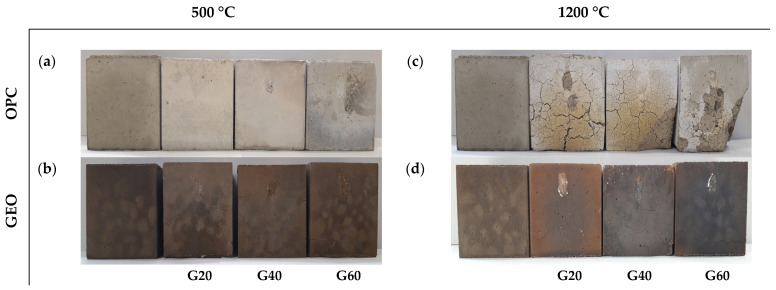
Visual observation of concrete in cm/cube: (**a**) OPC at 500 °C; (**b**) GEO at 500 °C; (**c**) OPC at 1200 °C; (**d**) GEO at 1200 °C.

The physical appearance of the specimens, for all standard grades of strength, before and after the exposure to fire, is shown in Figure 3. After the exposure to fire at 500 °C, a change in the color of the OPC-based concrete specimens (to white) was apparent. On the other hand, the geopolymer concrete specimens did not show any change in color. Minor cracks were visible on the surface of each specimen, with the high-strength concrete showing more cracks than the medium- and low-strength concretes. 

Subsequent to the exposure to fire at 1200 °C, apparent changes in color were detected for all specimens. For the OPC-based concrete specimens, the color of burnt specimens changed to whitish yellow, owing to the complete loss of moisture in the concrete. For the geopolymer concrete specimens, the color changed to reddish brown for the low-strength concrete and black for the medium- and high-strength concretes, owing to the high content of iron oxide in fly ash. Similar changes in color were also observed by Kong and Sanjayan [12], who performed the fire resistance evaluation on geopolymer composites. 

Surface cracking was prominent due to the high temperature differential between the surface and core of the specimens. Cracks were more evident on the OPC specimens than on the geopolymer concrete specimens, due to the complete loss of moisture within the concrete. 

Subsequent to the exposure to fire at 1200 °C, no spalling was observed on OPC20, as shown in Figure 3c. Spalling was more visible on OPC40 and OPC60. The spalling resulted from thermal stresses gained from the rapid increase in temperature and the differential internal pore pressure within the concrete during the exposure to fire. High pore pressure could not escape the concrete and, consequently, induced tensile stress in the concrete, resulting in spalling with the release of an explosive sound. Spalling of OPC60 specimens even resulted in the loss of cross-sectional area, which reduced the load capacity. 

Conversely, the geopolymer concrete specimens showed no spalling. Even though the structure of geopolymer concrete is denser than that of OPC-based concrete, the differential thermal stress did not cause spalling. Cracks on the surface of the specimens were evident subsequent to the exposure to fire at 1200 °C, but they were not predominant as compared to those on the OPC-based concrete specimens, as displayed in Figure 3d. According to the visual observations, the geopolymer concrete had a higher endurance against fire than the OPC-based concrete.

### 3.2. Mass Loss

**Figure 4:**
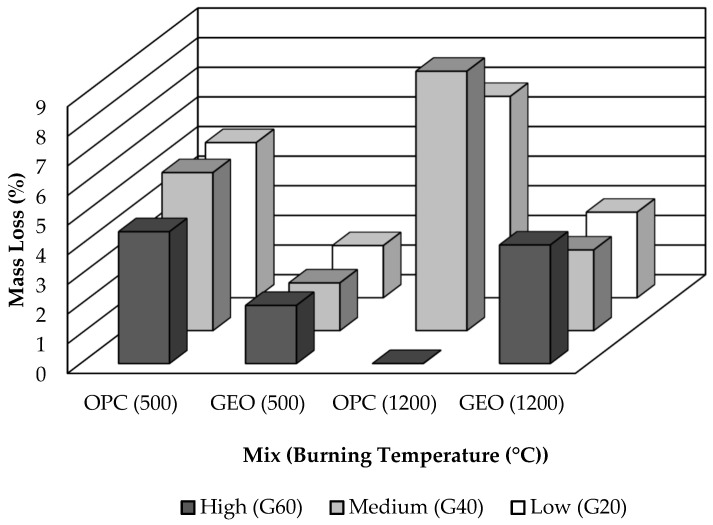
Mass losses of specimens after the exposure to fire at 500 and 1200 °C.

Mass loss refers to the mass of the burnt specimens that is expressed as a percentage of their original mass at ambient temperature. Mass losses of the specimens were determined by measuring the masses of the specimens before and after the exposure to fire. Figure 4 illustrates the effects of the exposure to fire on the mass loss. Evaporation of free water in the concrete matrix, along with spalling, caused significant damage to the specimens, resulting in mass loss [13]. The OPC-based concrete specimens evidently endured a higher mass loss than the geopolymer concrete specimens. Subsequent to the exposure to fire at 500 °C, OPC40 endured a mass loss of 5.332%, while GEO40 endured the lowest mass loss of 1.610%. As for the GEO20 and GEO60 specimens, the mass losses were also low, at 1.759% and 1.961%, respectively. OPC-based concrete suffered more than 4% mass loss, which is relatively high. 

The mass loss increased after the exposure to fire at 1200 °C. The mass losses were higher for OPC-based concrete than for geopolymer concrete, owing to breakdown of the hydration product and total loss of free water. When the concrete was exposed to fire at a high temperature, the free water evaporated. Volume of the evaporation rose as the heating temperature increased. Moreover, the OPC-based concrete suffered major cracks and spalling. OPC20 and OPC40 suffered high mass losses of 6.795% and 8.756%, respectively. As for OPC60, due to the significant loss in cross-sectional area from the spalling, its mass loss could not be determined, and is displayed as 0%. 

For the geopolymer concrete specimens, their mass losses were significantly lower than those of the OPC-based concrete specimens. GEO20 and GEO40 endured mass losses of 2.855% and 2.722%, respectively. The highest mass loss was that of GEO60 after the exposure to fire at 1200 °C, which was 4.001%. According to Kong and Sanjayan [12], the denser the matrix of the concrete, the higher the possibility that spalling will occur as heat stresses induce the internal pore pressure [12]. However, despite the fact that the geopolymer concrete matrix is substantially denser than the OPC-based concrete, it has a higher capacity to tolerate the internal pore pressure, due to the heat differential within the concrete, as substantiated by the absence of spalling and lower mass loss of the geopolymer concrete relative to the OPC-based concrete. Similar observations for geopolymer concrete were found by Ali [13] and Sarker et al. [14].

### 3.3. Residual Compressive Strength

**Figure 5:**
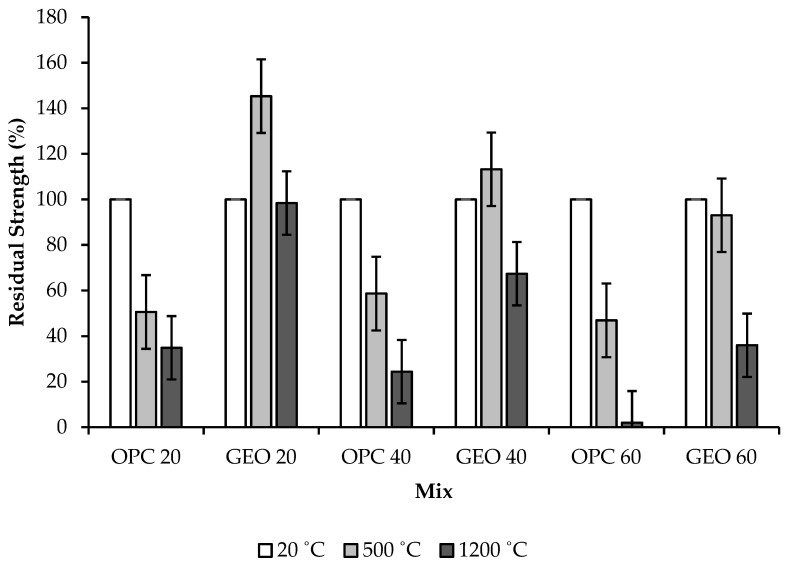
Residual compressive strength of concrete specimens.

The development of compressive strength was monitored for 28 days. Under the exposure to fire, the concrete gradually lost strength as the temperature increased. Figure 5 shows the residual compressive strength obtained from the specimens before and after the exposure to fire at 20 (or ambient temperature), 500, and 1200 °C. All OPC-based concrete specimens endured a rapid reduction in compressive strength after the exposure to fire as the burning temperature increased from 20 to 500 and 1200 °C. 

Most of the geopolymer specimens also suffered a reduction in strength, albeit at a slower rate than that of OPC-based concrete specimens. Conversely, GEO20 and GEO40 gained strength after the exposure to fire at 500 °C, with residual compressive strengths of 145.33% for GEO20 and 113.21% for GEO40. In addition, GEO60 lost strength by a small amount after the exposure to fire at 500 °C, with a residual compressive strength of 93.02%. For OPC-based concrete specimens exposed to fire at 500 °C, OPC40 obtained the highest residual compressive strength of 58.66%, while those of OPC20 and OPC60 were 50.6% and 46.93%, respectively. The increase in strength of geopolymer concrete after the exposure to fire demonstrated that, during the exposure to fire, further geopolymerization reactions took place in the concrete matrix. A similar increase in the strength of geopolymer concrete was also found by Kodur and Phan [15] and Sarker et al. [14].

The residual compressive strengths of all specimens decreased considerably after the exposure to fire at 1200 °C in comparison to those at 500 °C. GEO20 obtained a residual compressive strength of 98.4%, which was higher than those of GEO40 and GEO60, which obtained residual compressive strengths of 67.36% and 36%, respectively, indicating that low-strength geopolymer concrete possesses a high level of fire resistance. Moreover, after the exposure to fire, GEO40 was able to preserve more than 60% of the initial strength. Conversely, OPC-based concrete specimens suffered major losses in strength after the exposure to fire at 1200 °C; the residual compressive strengths were 34.9% for OPC20, 24.4% for OPC40, and 0% for OPC60.

The severe loss in strength of OPC-based concrete was facilitated by the total loss of water from evaporation, followed by the formation of major cracks and the occurrence of spalling due to the thermal stresses within the concrete. The high thermal strain within the concrete also contributed to the loss in strength. The higher reduction in strength of the OPC-based concrete relative to that of the geopolymer concrete is consistent with the findings of Ali [13], Sarker et al. [14], and Zhu and Wu [16], who mentioned that the OPC-based concrete disintegrated due to complete dehydration and spalling. On the other hand, the high residual compressive strength of geopolymer concrete suggests that its fire resistance is high, and that its structural integrity and stability were maintained, even after the exposure to high temperatures.

### 3.4. Microstructural Analysis

#### 3.4.1. Scanning Electron Microscopy (SEM)

**Figure 6:**
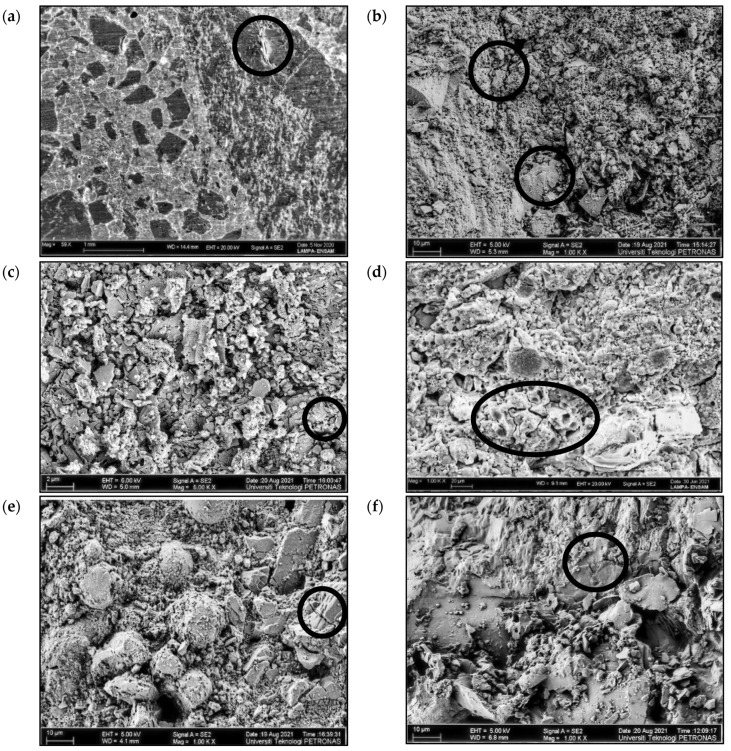
SEM images of (**a**) OPC40 at 20 °C; (**b**) OPC40 at 500 °C; (**c**) OPC40 at 1200 °C; (**d**) GEO40 at 20 °C; (**e**) GEO40 at 500 °C; (**f**) GEO40 at 1200 °C.

SEM analysis was performed on medium-strength specimens, namely, OPC40 and GEO40. SEM images of the specimens are presented in Figure 6. The images of OPC40 after the exposure to fire at 20, 500, and 1200 °C are shown in Figure 6a–c, respectively, while those of the GEO40 are shown in Figure 6d–f, respectively. The microstructure of GEO40 became denser with the increase in burning temperature to 1200 °C. Smaller amounts of unreacted fly ash are visible in GEO40 after the exposure to fire at 500 °C, as shown in Figure 6e. No new products resulting from the burning were observable on the surfaces of fly ash particles—only the formation of minor cracks. As the burning temperature increased to 1200 °C, the pores within the matrix of GEO40 started to connect, and fewer unreacted fly ash particles were observed. Furthermore, internal microstructural cracking was detected, as shown in Figure 6f. As for the OPC40 at 20 °C, the matrix was homogeneous, with the presence of fine cracks, as shown in Figure 6a. When exposed to fire at 500 °C, the matrix of OPC40 started to crumble and disintegrate, as shown in Figure 6b. Heavy damage to the specimen was observed after the exposure to fire at 1200 °C, as shown in Figure 6c, due to the complete dehydration, where brittle and crumbling cement paste was visible and a porous structure was created.

Differences in the matrix structures of OPC40 and GEO40, due to the exposure to fire, are evident. The matrix of OPC40 suffered heavy damage, with visible breaking of the bonds between the cement paste and aggregates. In contrast, the matrix of GEO40 appeared to be stable, homogeneous, and dense, at a high magnification level, after the exposure to fire, due to sintering and further geopolymerization of fly ash with the alkaline solution within the matrix, which is consistent with the findings of Kong and Sanjayan [6,12]. Loss of strength in geopolymer concrete specimens at higher temperatures was due to thermal shocks and incompatibility of the matrix with the aggregates. Eventually, geopolymer concrete specimens suffered a loss of strength, although it was not as significant and rapid as in OPC-based concrete specimens.

#### 3.4.2. Fourier-Transform Infrared Spectroscopy (FTIR)

**Figure 7:**
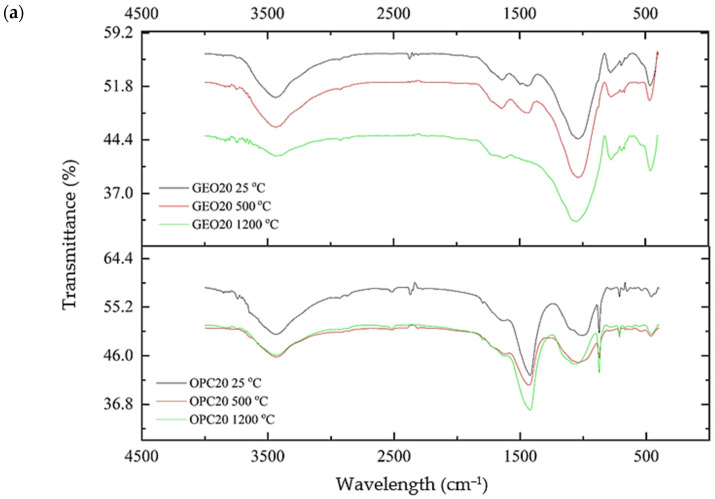

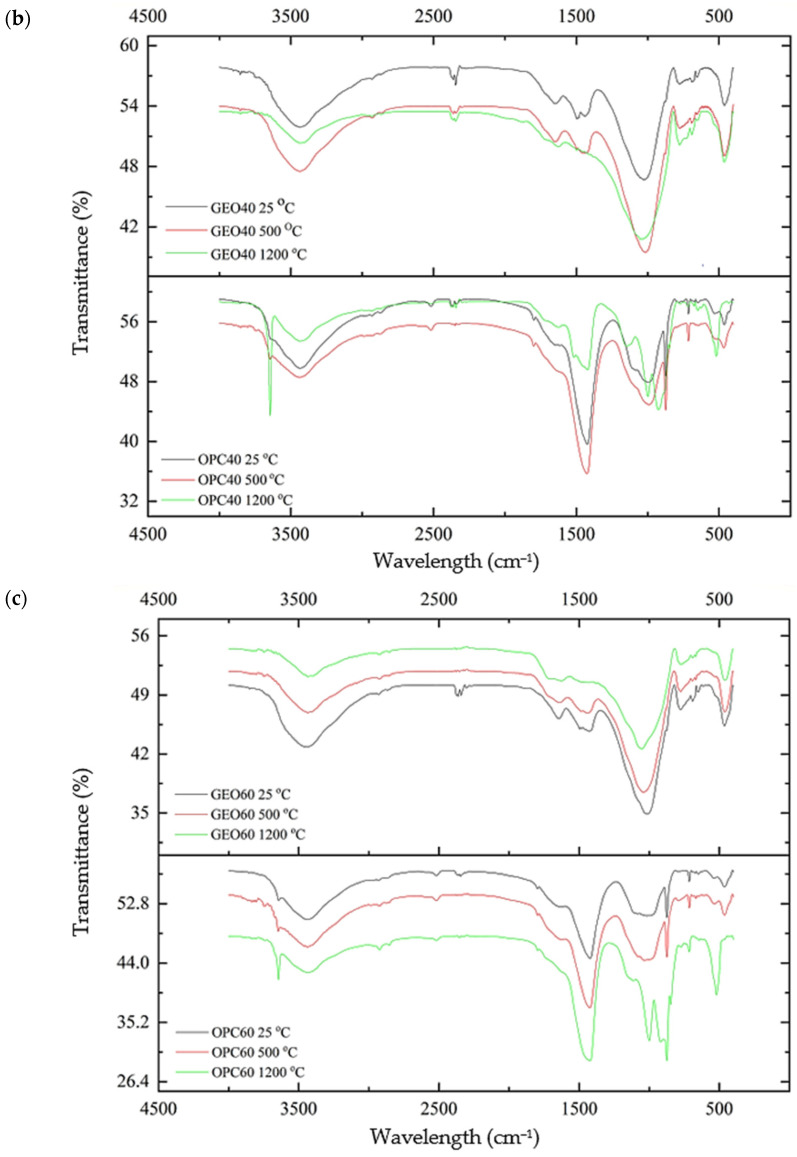
FTIR spectra of OPC-based and geopolymer concrete specimens of standard strength grades of (**a**) 20 MPa, (**b**) 40 MPa, and (**c**) 60 MPa.

FTIR analysis was conducted to characterize the specimens and indicate the pattern of chemical reaction changes. FTIR spectra of the specimens after the exposure to fire at 500 and 1200 °C are shown in Figure 7. The 1000 cm^−1^ band indicates that formation of geopolymers was taking place, where the transition of SiO_2_ and Al_2_O_3_ occurred due to the chemical reaction between fly ash and an alkaline solution. When the concrete was exposed to fire, there was a reduction in the intensity of the spectra. The wavelength number remained the same for the peak showing the corresponding T-O-T (T is tetrahedral Si or Al) group, due to the asymmetric stretching vibration. The low frequency also indicates that geopolymerization took place when the specimen was exposed to fire. Bands at 3500 and 1100 cm^−1^ decreased in intensity with increasing burning temperature, signifying the dehydration of geopolymers due to bending vibrations of the H-O-H and hydroxyl functional groups. The changes occurred due to the loss of bound water within the geopolymer concrete matrix [17]. Meanwhile, for OPC-based specimens, the bands at 900, 1400, and 3500 cm^−1^ represent the silicates in cement [18], and temperature increases shifted the bands near 800 and 1000 cm^−1^. The lower intensity indicates that the decomposition of C-S-H showed apparent changes in the OPC-based specimens. Comparable shifts for geopolymer and OPC-based concrete have been reported by Luhar et al. [19], indicating that the geopolymer concrete has a high thermal stability in comparison to OPC.

## 4. Conclusions

The fire resistance of fly-ash-based geopolymer concrete was evaluated relative to that of OPC-based concrete. Specimens were exposed to fire at 500 and 1200 °C for 2 h to simulate real fire events. Visual observation was performed on the physical appearance of the specimens before and after the exposure to fire in order to detect the formation of cracks and the occurrence of spalling. Mass loss and residual compressive strength of the specimens were measured before and after the exposure to fire. Microstructural analyses, comprising SEM and FTIR analyses, were also conducted. Based on the results obtained, the following conclusions can be drawn:

(a) OPC-based concrete suffered major cracks accompanied with spalling for the high-strength specimen, while geopolymer concrete experienced minor cracks with no spalling. Physical damage endured by the geopolymer concrete was minor compared to that endured by the OPC-based concrete. Therefore, the structural integrity of the geopolymer concrete was maintained after the exposure to fire;
(b) Mass losses of the OPC-based concrete were 5.33% and 8.75%, while those of the geopolymer concrete were 1.69% and 4%, after the exposure to fire at 500 and 1200 °C, respectively, due to dehydration of free water within the concrete. Therefore, the geopolymer concrete demonstrated a higher level of fire resistance in comparison to the OPC-based concrete;
(c) Measurements of residual compressive strength revealed that the geopolymer concrete displayed excellent stability and fire resistance. More than 50% of the residual compressive strength for low- and medium-strength geopolymer concrete was maintained after the exposure to fire at 1200 °C. Subsequent to the exposure to fire at 500 °C, the residual compressive strength of the geopolymer concrete increased from 13% to 45%, while the OPC-based concrete was not able to sustain its compressive strength, due to complete dehydration of the concrete;
(d) SEM images showed that the matrix of the geopolymer concrete, at the microstructural level, is denser than that of the OPC-based concrete after the exposure to fire, owing to further geopolymerization reaction during the exposure to fire. The OPC-based concrete endured heavy damage due to the decomposition of cement paste and disintegration of cement–aggregate bonding;
(e) The FTIR spectra of the geopolymer concrete showed a minor shift in wavelength due to the loss of water, and low intensity owing to chemical reactions that occurred during the exposure to fire, indicating that the geopolymer concrete has excellent thermal stability after the exposure to fire.

The findings of this study confirm that fly-ash-based geopolymer concrete has excellent fire resistance in comparison to OPC-based concrete. Analyses of the heat transfer and thermal distribution of geopolymer concrete with the exposure to fire, as well as investigations that consider the influence of the distance between the flame and the specimen, are recommended for future research.

## Figures and Tables

**Table 1:** Elemental compositions of the ordinary Portland cement (OPC) and fly ash.

Oxides (%)	SiO_2_	Al_2_O_3_	Fe_2_O_3_	CaO	SO_3_	K_2_O	TiO_2_	SrO	P_2_O_5_	Loss on Ignition
OPC	20.06	4.93	2.86	63.94	3.67	-	-	-	-	1.45
Fly Ash	75.64	12.04	3.36	2.35	1.5	2.02	1.20	0.1	1.7	2.36

**Table 2:** Concrete mix proportions.

*OPC-Based Concrete*										
**Mix**	**OPC** **(kg/m^3^)**		**Water** **(kg/m^3^)**		**Sand** **(kg/m^3^)**		**Coarse Aggregates** **(kg/m^3^)**		**Superplasticizer** **(kg/m^3^)**	
OPC20	342		205		652		1211		-	
OPC40	405		190		642		1193		-	
OPC60	600		190		498		1162		4	
*Geopolymer Concrete*										
**Mix**	**Fly Ash** **(kg/m^3^)**	**Sand** **(kg/m^3^)**		**Coarse Aggregates** **(kg/m^3^)**		**Sodium Hydroxide** **(kg/m^3^)**		**Sodium Silicate** **(kg/m^3^)**		**Water** **(kg/m^3^)**
GEO20	400	850		950		57		143		40
GEO40	400	640		1000		43.5		108.5		40
GEO60	460	700		1050		46		138		46

## Data Availability

All data are contained within the article.
